# Correlation of serum adiponectin with thyroid profile among metabolic syndrome patients with and without hypothyroidism

**DOI:** 10.6026/973206300212971

**Published:** 2025-09-30

**Authors:** Selvakumar D., Pavana A., Nalugotla Lakshmanna, Gnanadesigan Ekambaram, Veluri Ganesh

**Affiliations:** 1 Department of Biochemistry, PES Institute of Medical Sciences and Research, Kuppam, Chittoor, Andhra Pradesh, India-517425; 2 Department of Biochemistry, Sri Siddhartha Institute Of Medical Sciences, T Begur, Karnataka, India-562123; 3 Department of Biochemistry, Kurnool Medical College, Kurnool Andhra Pradesh, India-518002; 4 Department of Physiology, Nootan Medical College and Research Centre, Sankalchand Patel University, Visnagar, Gujarat, India- 384315; 5 Department of Biochemistry, PES University Institute of Medical Sciences and Research, Electronic City, Bengaluru, Karnataka, India-560100

**Keywords:** Adiponectin, ferric reducing ability of plasma, malondialdehyde, metabolic syndrome

## Abstract

The Metabolic Syndrome is becoming a more significant global public health condition, and look for reliable, sensitive biomarkers that may be used as diagnostic tools to identify metabolic syndrome. Therefore, it is of interest to correlate the serum adiponectin with thyroid profile in metabolic syndrome patients with and without hypothyroidism. This is a cross-sectional study that included total 120 metabolic syndromes with and without hypothyroidism and 30 healthy controls. The analysis of biochemical and experimental parameters was done. The serum adiponectin was significant and drastically decreased levels was observed in metabolic syndrome patients with and without hypothyroidism and healthy controls (P = 0.001**). It was observed that serum adiponectin was negatively correlated with blood sugars, lipid profile, MDA, TSH and positively correlated with FRAP (P = 0.001**). Thus, we show significantly decreased levels of serum adiponectin among metabolic syndrome patients with and without hypothyroidism might be useful for identify complications like type 2 diabetes mellitus and cardiovascular diseases.

## Background:

Metabolic Syndrome (Met S) is becoming a more significant global public health condition and the risk factors of it are obesity, dyslipidemia, increased blood pressure and blood sugars [1-2]. Recent studies are reporting that the Met S is a pandemic affecting a wide range of people, so it is imperative to look into the mechanisms underlying the condition and look for reliable, sensitive biomarkers that may be used as diagnostic tools to identify metabolic syndrome [3-4]. According to the International Diabetic Federation, central obesity, additional two or more risk factors were used for diagnosis of Met S [5]. Since obesity is becoming an epidemic on a global scale, it has been observed that almost 1.4 billion people have Met S. The International Diabetic Federation (IDF) estimates that 25% of the world's population has Met S [6- 7]. In the western population, the prevalence of Met S in adults is approximately 20%. In India, the study population's Met S prevalence is 23.2% based on the WHO, 18.3% in relation to the NCEP: ATP III therefore, and 25.8% according to the IDF definition [8]. Met S has been linked to thyroid malfunction, specifically subclinical hypothyroidism (SCH), in numerous epidemiological studies. According to research, thyroid hormone has a crucial role in the proliferation and differentiation of adipocytes, two processes that are necessary for adipocytes to operate normally physiologically [9-10]. The regulation of adipocytes' release of adipokines is linked to normal thyroid function. Consequently, any change in thyroid function may result in anomalies in adipokine homeostasis and consequently related comorbidities such as type II diabetes in Met S. It is currently uncertain what precise mechanism connects type II diabetes, Met S and thyroid disorders [11-12]. Adiponectin is the hormone synthesized from adipose tissue and involved in improves insulin sensitivity, act against atherogenic and inflammatory molecules [13]. It is involved in carbohydrate, lipid metabolism and also plays an essential role in homeostasis of cardiac muscles [14]. Increased obesity leads to decreased levels of adiponectin resulting in insulin resistance, improper functions of thyroid gland in Met S patients [15]. Therefore, it is of interest to analyse the Correlation of serum adiponectin with thyroid profile in metabolic syndrome patients with and without hypothyroidism.

## Materials and Methods:

This is a cross-sectional study which was conducted in the Department of Biochemistry, in collaboration with the Department of Medicine, at PES University Institute of Medical Sciences, Bangalore, India from Sep 2024- June 2025. Approval from the Institutional Ethics Committee (IEC) was obtained, and informed consent was collected from all respondents. A total of 60 patients with metabolic syndrome were diagnosed according to IDF criteria and divided into two groups on the basis of the presence or absence of hypothyroidism. Additionally, 30 healthy controls, were included in the study (who were matched for Body Mass Index (BMI), gender and age) shown in Table 1 (see PDF).

## Inclusion criteria:

All participants were required to be between 30 and 70 years of age. The healthy controls were free from any illnesses, and the metabolic syndrome (Met S) patients were diagnosed following the IDF guidelines. The thyroid stimulating hormone greater than 8 µIU/mL considered as hypothyroidism. 

## Exclusion criteria:

The study excluded pregnant and lactating women, individuals with acute or chronic infectious diseases, liver or kidney diseases, cardiac conditions, hyperthyroidism, urinary tract infections, and those unwilling to participate. Blood sample collection: A fasting blood sample of five (5) milliliters (mL) was collected from each participant and distributed as follows: 1 mL into a fluoride tube, 2 mL into an Ethylene Diamine Tetra Acetic Acid (EDTA) tube, and the remaining 3 mL into a plain tube. Plasma and serum were separated through centrifugation, and the resulting aliquots were properly labeled and stored at -50°C until analysis. Routine laboratory tests like fasting blood sugar (FBS), triglycerides (TGL), total cholesterol (TC), and high-density lipoprotein (HDL), were performed using standard laboratory methods. Glycated hemoglobin (HbA1c) was measured by the immunoturbidometric method, while serum malonaldehyde (MDA) and ferric reducing ability of plasma (FRAP) were assessed using commercially available kits. Total T4, Total T3, adiponectin levels, and thyroid-stimulating hormone (TSH) were determined through Enzyme-Linked Immunosorbent Assay (ELISA) using the Chem Ultra-Euro Fully automatic analyzer, Mindray CL-1200i, and Immuno Assay Automatic Analyzer.

## Statistical analysis:

Kolmogorov-Smirnov test was used to analyse the distribution of data, which was then expressed as mean ± standard deviation (SD). Analysis of variance (ANOVA) was used for comparisons between groups, while the correlation between serum adiponectin and other variables among the study subjects was analyzed using Pearson's correlation. A p value of less than 0.05 was considered to be statistically significant. Statistical Package for the Social Sciences (SPSS) and Microsoft Office Excel were used for all statistical analyses.

## Results:

Table 2 (see PDF) illustrates clinical, biochemical, and demographic characteristics of the participants. The weight, height, age, Diastolic Blood Pressure (DBP), Systolic Blood Pressure (SBP), and Body Mass Index (BMI) were significantly high in subjects with Met S as compared to controls (P = 0.001**). The mean ± SD of FBS, PPBS, Lipid profile were significantly elevated in Met S as compared to controls (P = 0.001**). The thyroid profile showed statistical significance as compared to controls (P =0.001**). Additionally, we also found MDA and adiponectin levels was significantly higher in Met S patients as compared to controls (P = 0.001**) (Figure 1 - see PDF). Furthermore, The FRAP and adiponectin levels was significantly decreased in Met S with hypothyroidism and without hypothyroidism and controls (P = 0.001**) (Figures 2 - see PDF and 3 - see PDF). The age, height, weight, BMI, SBP and DBP shows the significantly elevated levels in patients with Met S with hypothyroidism and without hypothyroidism as compared to healthy controls (P = 0.001**). The Met S patients with hypothyroidism shown very high significant levels of FBS, PPBS, TGL, TC, HDL, VLDL, and LDL when compared to Met S without hypothyroidism and controls (P=0.001**). The TSH levels were also significantly elevated in patients with Met S with hypothyroidism than Met S without hypothyroidism and controls (P = 0.001**). No significant levels of FRAP, T3, and T4 were found between the study groups (P=0.764, 0.954 and 0.221). Additionally, we found serum MDA drastically elevated in Met S with hypothyroidism, Met S without hypothyroidism and controls (P = 0.001**). Furthermore, we observed significantly decreased levels of serum adiponectin in Met S with hypothyroidism, Met S without hypothyroidism and controls (P = 0.001**) (Table 3 - see PDF).

A statistically significant increase in weight, height, BMI, SBP, DBP, between the Met S with and without hypothyroidism and controls (P=0.001**) can be seen in Table 4 (see PDF) and age is not shown significant between the Met S without hypothyroidism and Met S with hypothyroidism (P=0.190). The blood sugars and lipid profile shown high significant between Met S without hypothyroidism and controls (P=0.001**) and there was no significance between Met S with and without hypothyroidism (P>0.05). Additionally, T3, T4 and FRAP levels also not shown any significance between Met S with and without hypothyroidism and controls (P=0.001**). There was a statistically significant increased levels of MDA and adiponectin in Met S with and without hypothyroidism compared to controls (P=0.001**). Pearson's correlation analysis done between the serum adiponectin and other various variables of the study shown in Table 5 (see PDF). The serum adiponectin was found to have significant negative correlation with BMI, FBS, PPBS, TGL, TC, VLDL, LDL, MDA, TSH (P = 0.001**) and significant positive correlation with HDL (0.001**). Additionally, we also observed there is the adiponectin shown no significant negative correlation with T3, T4 was observed.

## Discussion:

The metabolic syndrome, which is typified by a series of disorders related to metabolism, such as insulin resistance, dyslipidemia, hypertension, and central obesity, enhances the risk of developing type II diabetes mellitus and atherosclerotic cardiovascular illnesses [16]. Three or more of these metabolic anomalies are required for the diagnosis of metabolic syndrome, indicating the critical need for early detection and intervention techniques [17]. The over the last twenty years, there has been a noticeable rise in the prevalence of metabolic syndrome. The significant elevation of Met S between 20 and 25 percent of people worldwide [18]. The Met S patients because adipocytes and thyroid lead many complications such as hypothyroidism, type 2 diabetes mellitus and cardio vascular diseases [19]. There is a significant overlap among the two most prevalent endocrine illnesses, thyroid dysfunctions and the metabolic syndrome. Due to their strong associations with morbidity and mortality, both have a huge global influence on health care [20]. The peripheral alterations that directly affect body weight management, the altered metabolism of lipids and glucose, and the control of blood pressure that are observed in metabolic syndrome are intricately linked to the previously stated central abnormalities in autonomic regulation [21]. There have been reports of elevated levels of thyroid stimulating hormone (TSH) in individuals with metabolic syndrome compared to healthy individuals, as well as a higher incidence of metabolic syndrome in individuals with elevated TSH levels relative to those with normal TSH levels [22- 23].

In this study we found significant increased levels of SBP, DBP, fasting blood sugars, total cholesterol, triacyl glycerides, very low-density lipoproteins, low density lipoproteins, and decreased high density lipoproteins in patients with Met S with hypothyroidism when compared to Met S without hypothyroidism and controls. Similarly, previous studies also reported that significantly increased levels of blood pressure, fasting blood sugar, dyslipidemia in Met S with and without hypothyroidism when compared to controls [24, 25-26]. The present study also observed significantly elevated levels of MDA in patients Met S with and without hypothyroidism when compared to controls. The Previous studies also observed significantly increased levels of serum MDA levels in metabolic syndrome and healthy controls [27-28]. The serum FRAP levels also significantly reduced in Met S with and without hypothyroidism when compared to controls. Similarly, recent studies also found significantly decreased levels of total antioxidants (FRAP) in patients with and without Met S and controls [29, 30-31]. Adiponectin is an adipocytokine has 247 amino acids and it exhibit in circulation 3 distinct forms low, middle and high molecular weight. Recent studies are reported that high molecular weight adiponectin associated with Met S, T2DM and cardiovascular diseases based on its anti-inflammatory, antioxidative and anti-atherogenic properties [32]. In the present study we found thyroid dysfunction in patients with Met S and also shown there was a significantly decreased levels of serum adiponectin when compared to Met S without hypothyroidism and controls. A significant negative correlation was identified between adiponectin and fasting blood sugars, lipid profile, blood pressure in Met S patients with hypothyroidism. Similarly, the previous studies also reported significantly decreased levels of serum adiponectin and these levels of negatively correlated with blood sugars, dyslipidemia and blood pressure [32, 33-34]. The decrease levels of serum adiponectin beneficial for the metabolic patients with and without hypothyroidism.

## Conclusion:

Significantly reduced levels of serum adiponectin might be useful for to identify complications among metabolic syndrome patients. Thus, we show continuous monitoring of serum adiponectin along with the other parameters useful for metabolic patients with and without hypothyroidism is needed.
